# Land-Scurvy

**Published:** 1847-07

**Authors:** 


*Land-Scurvy.*—We understand that land-scurvy is becoming prevalent in various parts of the kingdom. A great many many cases, with the features resembling those of sea-scurvy well marked, have been lately brought into the Edinburgh Infirmary. The patients had been labourers on railways, living on bad diet, and working on moors far from villages, so that they were not able to procure milk or vegetables, or even the common conveniences for cooking their food. It is singular that, owing as it appears to the great dearth of vegetable food, a disease which has been long extinct in the navy, is now making its appearance on land. The deaths from purpura registered last week in the metropolis were 5. against a spring average of 0.4. *Lond. Med. Gaz.*

